# Editorial: Using Cancer ‘Omics’ to Understand Cancer

**DOI:** 10.3389/fonc.2020.01201

**Published:** 2020-07-24

**Authors:** Barbara K. Dunn, Daoud Meerzaman

**Affiliations:** ^1^Division of Cancer Prevention (NCI), Bethesda, MD, United States; ^2^Center for Biomedical Informatics and Information, Technology (NCI), Rockville, MD, United States

**Keywords:** big data, cancer genomics, cancer genomics data analysis, HPC (high performance computing), humanizing big data

The notion of using the “big data” approach to study human disease is not new. Scientists have been tapping data from studies of genomics, proteomics, transcriptomics, metabolomics, and microbiomics since the initial mapping of the human genome (1). What has changed, however, is a fundamental shift in how we think about these technologies. The “omics” field is expanding in scope, blending biology, technology (radiomics), and clinical observations (electronic health records), as well as size. This amplification of content and quantity has required parallel development and application of novel informatic tools. The need to accommodate the ever-larger datasets critical to our understanding of cancer omics has instigated a movement toward development of high-performance computing, including both hardware and software to analyze the massive, generated big data. The manuscripts contained in this volume reflect this constantly evolving panel of bioinformatic programs and resources with capacity to carry out large-scale data analysis.

Most of the papers in this issue report findings that share the common feature that all distill a select number of biomarkers from a large spectrum of potential markers from an analysis of large datasets. This volume of Frontiers broadens its approach to include papers dealing directly with the attributes, management, and clinical application of big data. Focusing on some of the key databases, projects and methodologies developed to implement such analyses, emphasizing the ever-expanding scale of big data, exascale computing is discussed. At the initiation of marker discovery, the patients and other individuals who serve as the source of big data are highlighted, while encouraging big data researchers to keep in mind the humanity inherent in these data (Helzlsouer et al.).

To date much of our focus has been on comparing the omics information of cancer patients with that of “normal” controls, i.e., healthy individuals, and looking for genotypic or phenotypic differences that set the patients apart. This rudimentary approach has led to practical applications, including offering targets for early detection, prognosis, and treatment. Along these lines, in this special issue of Frontiers, several authors address genomic (and epigenomic) abnormalities that characterize specific cancers and may thus have practical applications at the clinical level.

The manuscript by Yang et al. offers an example of the application of omics research to biomarker discovery. This paper describes potential diagnostic markers and therapeutic targets for leiomyosarcoma (LMS). This cancer is particularly aggressive, with invasive clinical characteristics and often a poor prognosis. Finding new biomarkers to assess malignancy and prognosis of LMS is critical. Yang et al. used Weighted Gene Co-expression Network Analysis (WGCNA), a systematic molecular clustering approach, to look for gene expression patterns that are associated with LMS and thereby should help to improve our understanding of the molecular mechanisms of this cancer. Their results showed that the expression of CDK4, CCT2, and MGAT1 in LMS tissues was significantly higher than that in adjacent tissues, suggesting that these genes may be part of the cancer signaling pathway. Such findings could pave the way for new strategies for diagnosing and treating LMS.

Another cancer, glioblastoma multiforme (GBM), is the focus of two articles in this special volume, by Cheng et al. and by Stajkovska et al. Cheng et al. employed a data mining approach by tapping into The Cancer Genome Atlas (TCGA). TCGA is managed by the Genomic Data Commons (GDC) (2) funded by the National Cancer Institute (NCI) which provides the cancer research community with a unified data repository that enables data sharing across cancer genomic studies in support of precision medicine. They then applied various bioinformatic tools aimed at discovery of relevant genes and pathways. They examined the gene expression patterns of transcription factors associated with GBM and identified four potential candidates based on their differential expression between tumor and adjacent tissue: *LHX2, MEOX2, SNAI2*, and *ZNF22*. By clustering transcription factors that are differentially expressed in GBM and screening these clusters using appropriate bioinformatic programs, they identified cancer pathways primarily associated with cell migration, cell adhesion, epithelial-mesenchymal transition (EMT), cell cycle, as well as other signaling pathways. Combining these results with patient characteristics, such as risk score, age, gender, type of treatment, and treatment response, these authors showed that their model was able to precisely predict the outcome of patients with GBM. GBM was further explored in the study by Stajkovska et al., in their description of a case report of a pediatric patient. Using targeted gene panel testing in blood and tumor tissue, these researchers identified a heterozygous frameshift mutation (c.333_334delTC; p.His112CysfsTer9) in the *MLH1* gene in addition to a known heterozygous missense variant of unknown significance/VUS (c.847C > T; p.Arg283Cys) in the *TP53* gene. Screening of the patient's parents revealed the presence of the *MLH1* abnormality in the father and the *TP53* variant in the mother. They report for the first time the co-occurrence of a genetic mutation in the *MLH1* gene of the mismatch repair pathway, often associated with Lynch syndrome, accompanied by a rare variant in the *TP53* gene. The authors stress that co-occurrence of multiple gene abnormalities should be considered as a possible contributory cause of a cancer. However, caution must be exercised in interpreting a VUS as contributing to the cancer phenotype, as these variants are of unproven pathogenicity, a subject addressed in Helzlsouer et al. in this volume.

Biomarkers also are the focus of the study by Wang Y. et al., who looked at new ways of predicting the progression and prognosis of bladder cancer (BC) using a big data approach. Through a series of screenings and WGCNA they identified “hub” genes (i.e., a hub gene serves as the focal point of interaction with other genes; in general, the genes connected to the hub are critical to gene regulation and other biological processes). Gene-set enrichment analysis (GSEA) revealed that the sets of highly expressed hub genes were mainly enriched in “bladder cancer,” “cell cycle,” and “ubiquitin-mediated proteolysis” related pathways. They further honed their results to two genes (*ANLN, HMMR*), which had prognostic value for different stages and grades of BC. These genes not only could accurately predict the overall survival of patients with BC, but also the progression-free survival, a common outcome measure in clinical trials.

In another biomarker study included in this volume, Wang X. et al. showed how a set of small nucleolar RNAs (snoRNAs), which guide the modification of other RNAs and which have been implicated in alternative splicing, can predict overall survival of gastric cancer patients. An eight-snoRNA risk signature serves as a prognostic factor in gastric cancer. The authors validated the expression patterns of these eight snoRNAs, both in cell lines and patients' tissues. The authors point out that seven of these snoRNAs correlate with survival, suggesting relevance of these markers to the clinical behavior of the bladder cancer. One snoRNA, U66, was linked to cell proliferation. These findings provide potential prognostic and therapeutic clues into gastric cancer.

Nersisyan et al. addressed the mechanistic basis of tumorigenesis by examining the component that involves telomere status. Unlike normal cells where telomeres are shortened with each cell division, telomere maintenance mechanisms (TMMs) are found in most cancers. Of the two types of TMMs found in cancer, most cancers exhibit a TMM that is activated via the classical “telomerase” pathway (TEL), using the telomerase ribonucleoprotein, which contains an RNA template that guides the synthesis of the telomere DNA. In contrast, the alternative TMM, which operates in a smaller proportion of tumors, is the “alternative lengthening of telomeres” (ALT) pathway. The ALT pathway, which relies on complex molecular mechanisms including homologous recombination events between telomeric sister chromatid strands, occurs in the context of an altered chromatin environment at the telomere region. Nersisyan and colleagues compared the TMM pathways in colorectal cancers (CRC) with microsatellite instability/MSI (both CRCs in Lynch syndrome/LS-CRC and sporadic MSI CRCs/MSI s-CRC) to a subset of sporadic microsatellite stable (MSS) CRCs as well as benign mucosa. In their study of alterations of telomere length, sequence composition, and transcriptional regulation in relation to the two types of TMMs (TEL, ALT) in CRCs, they applied bioinformatic analysis to big data from whole genome DNA and RNA sequencing together with a pathway model. They observed transcriptomic signatures that distinguish the two TMM subtypes in CRC, with ALT-TMM being slightly more prominent in hypermutated MSI s-CRC and LS-CRC.

Chen et al. show how DNA methylation, an important regulator of gene expression, can be used, along with other tumor and patient characteristics, to identify glioma subgroups that exhibit specific prognostic features. DNA methylation patterns were examined in 653 gliomas from the TCGA database of NCI. The authors used consensus clustering to narrow their findings of methylation levels at each CpG site known to influence survival into five subgroups. DNA methylation patterns were then correlated with age, tumor stage, and prognosis. WGCNA of the CpG sites identified 11 clusters that could be used to differentiate between high- and low-methylation groups and which could be further used to determine prognostic information about the glioma patients. When applied to *in vitro* experiments, an inverse relationship was shown between methylation level of glioma cells and their ability to migrate or their inability to respond to standard glioma therapies, temozolomide or radiotherapy. Thus, epigenetic (methylation) subtypes could potentially serve as markers for prognosis as well as guides to glioma therapies.

Lee's article offers context to this study of methylation in carcinogenesis by providing an overview of epigenetics, highlighting how abnormal epigenetic modifications contribute to the development of cancer. Beyond reviewing the basic molecular mechanisms of epigenetic regulation of gene expression (methylation, histone modification, and non-coding RNAs), Lee discusses the role of epigenetics in regulating differentiation during development while simultaneously maintaining epigenetic memory during mitotic cell division. As an example, abnormal methylation of tumor suppressor genes downregulates expression, which when coupled with a mutation in the other allele contributes to carcinogenesis, according to the two-hit theory of Knudson. This concept is broadened to allele-specific gene expression (ASE) in general and its epigenetic regulation by allele-specific methylation (ASM). Starting from these descriptions of individual epigenetic abnormalities leading to cancer, the article extends into the epigenomic realm. Lee points out how the use of big datasets such as TCGA serve as a source not only of genomic information for analytic exploration but also for comparable investigations into large-scale epigenomic data. A prototypic example is the investigation of the TCGA dataset that identified a subset of GBMs with high CpG island methylation, subsequently labeled as a “glioma CpG island methylator phenotype” (G-CIMP). Clinical correlation of G-CIMP-positive tumors included higher prevalence among lower-grade gliomas and increased association with isocitrate dehydrogenase 1 (IDH1) somatic mutations. G-CIMP serves as merely one illustration of the extension of big data applications into the epigenetic, now the epigenomic, domain. This paper concludes by bringing the fruits of epigenetic/epigenomic research into the clinical realm, enumerating examples of approved cancer therapies that target cancer-inducing epigenetic abnormalities.

High throughput studies addressing big data also are helping us to identify subgroups of patients to better understand how disease affects certain populations. This approach has potential to predict which populations have patients who are more likely to respond to certain medications. Using lower throughput platforms, Kénémé and Sémbène studied genetic determinants of uterine fibroids (UF), benign tumors that are more frequent and are associated with more severe symptoms in African-American women. Focusing on 55 Senegalese women, their examination of genetic abnormalities in UFs in this population disclosed high genetic variability in repetition number of a GT dinucleotide microsatellite in the first intron of the C*OL1A2* gene. In addition to microsatellite instability, two GT sites had distinct mutations in the UFs in subsets of women. Furthermore, beyond confirming the involvement of the C*OL1A2* dinucleotide length polymorphism, GT_n_, in the occurrence of uterine fibroids in Senegalese women, these UF-associated genetic variants were additionally analyzed in relation to ethnicity, marital status, contraception use, diet, and physical activity. For the first time, these epidemiologic factors were shown to exhibit associations with the genetic underpinnings of UFs in this population. The authors consider that these results may create avenues for understanding the mechanisms involved in the racial variation in the prevalence and symptomatic severity of UFs as well as the predisposing factors.

The contents of this volume to this point have addressed the use of big data in investigations of various types of molecular mechanisms that underlie carcinogenesis in general and in specific cancers (and benign tumors). In contrast, Bhattacharya et al. delve directly into the nature and operation of data science, enumerating those attributes that enable its application to the discovery of carcinogenic mechanisms that are potentially targetable for prevention and treatment. The authors demonstrate how progress in the quantity and diversity of biomedical data, together with advances in artificial intelligence (AI) and machine learning (ML) algorithms, as well as computer architectures, enable advances in big data with a goal of accelerating cancer research. The authors take AI and ML to exascale levels, which are orders of magnitude higher than those of current high-end machines, in order to gain a deeper understanding of cancer. They describe a collaboration between the Department of Energy (DOE) and the NCI, the Joint Design of Advanced Computing Solutions for Cancer (JDACS4C), which has three pilot projects intended to push the frontiers of computing technologies in cancer research at the cellular, molecular and population levels. An example of the first pilot involves the application of exascale computing technology to a precision medicine initiative to develop predictive capabilities of drug response in pre-clinical models, ultimately leading to targeted cancer therapies in the clinic. The evolving needs of population databases, such as the Surveillance, Epidemiology, and End Results (SEER) registry of U.S. cancer incidence, as they increase the breadth of information collected, are being addressed by the high-performance computing and AI, as seen in the third pilot. The potential scope of applications of exascale computing is vast and multimodal, with potential for improving our understanding and management of cancer.

As evidenced by this special issue of *Frontiers in Oncology*, the omics field and the big data tools designed to support cancer research already are yielding results that are being translated into clinical practice. Helzlsouer et al. remind us, however, that the source of every piece of data is a human being. This connection must not get lost as we delve into the technical processes of sample collection, preparation, and analysis, both in the laboratory and at the informatic levels. In essence, we must take special care to “humanize” these big data. Helzlsouer et al. show that it is also critical to examine the challenges of genetic/genomic testing at the individual level, i.e., the human level. The limitations to clinical implications derived from analyses of big data, including the probabilistic nature inherent in genetic findings, need to be made clear to patients, but also to all health care providers. Maintaining the human aspect of these data sources is vital as we look to translate and apply findings to the cancer research field.

Today's big data require centralized, well-curated, and readily accessible databases that accommodate large-scale datasets. To this end, the National Institutes of Health and the NCI are actively contributing by establishing a number of data repositories within a larger Cancer Research Data Commons (CRDC) (3). These storehouses of data, coupled with large-scale, high-throughput sequencing technologies (genome, transcriptome, proteome), and deep machine learning, are resulting in exponential growth in data-driven solutions.

This special volume provides only a snapshot of articles featuring applications and approaches to omics data. Yet, this is an area that is just beginning to see its full potential. Big data are expanding our understanding of disease at its most fundamental level. The manuscripts in this special issue, given their diversity, reflect the multidisciplinary nature of the field. They further underscore the importance of collaboration using a fully integrated approach, from basic scientists to data/computational/modeling analysts (4).

We've come a long way since first mapping the genome. As we further unlock individual genomes we need to take care that we can protect personal information and avoid the potential for bias, highlighting the ethical aspect of data derived from humans Helzlsouer et al.. The use and reuse of data need to be carefully managed so that the interest and welfare of patients and others who share their data are maintained. In another decade we are sure to realize even greater advances in how we prevent, diagnose, and treat not only cancer, but a broad range of diseases, relying on the availability of robust big data.

## Author Contributions

BD and DM contributed to the conceptualization and writing the manuscript. All authors contributed to the article and approved the submitted version.

## Conflict of Interest

The authors declare that the research was conducted in the absence of any commercial or financial relationships that could be construed as a potential conflict of interest.

## References

[1] Hasin Y Seldin M Lusis A. Multi-omics approaches to disease. Genome Biol. (2017) 18:83. 10.1186/s13059-017-1215-1 28476144 PMC5418815

[2] Genomic Data Commons (GDC) Available online at: https://gdc.cancer.gov.

[3] Cancer Research Data Commons/CRDC Available online at: https://datascience.cancer.gov/data-commons.

[4] Meerzaman D Dunn BK. Value of collaboration among multi-domain experts in analysis of high-throughput genomics data. Cancer Res. (2019) 79:5140–5. 10.1158/0008-5472 31337654 PMC6801074

